# Structure-function clustering in weighted brain networks

**DOI:** 10.1038/s41598-022-19994-9

**Published:** 2022-10-06

**Authors:** Jonathan J. Crofts, Michael Forrester, Stephen Coombes, Reuben D. O’Dea

**Affiliations:** 1 Department of Physics and Mathematics, Nottingham Trent University, Nottingham, NG11 8NS UK; 2 School of Mathematical Sciences, University of Nottingham, Nottingham, NG7 2RD UK

**Keywords:** Complex networks, Phase transitions and critical phenomena, Dynamical systems, Network models

## Abstract

Functional networks, which typically describe patterns of activity taking place across the cerebral cortex, are widely studied in neuroscience. The dynamical features of these networks, and in particular their deviation from the relatively static structural network, are thought to be key to higher brain function. The interactions between such structural networks and emergent function, and the multimodal neuroimaging approaches and common analysis according to frequency band motivate a multilayer network approach. However, many such investigations rely on arbitrary threshold choices that convert dense, weighted networks to sparse, binary structures. Here, we generalise a measure of multiplex clustering to describe weighted multiplexes with arbitrarily-many layers. Moreover, we extend a recently-developed measure of structure-function clustering (that describes the disparity between anatomical connectivity and functional networks) to the weighted case. To demonstrate its utility we combine human connectome data with simulated neural activity and bifurcation analysis. Our results indicate that this new measure can extract neurologically relevant features not readily apparent in analogous single-layer analyses. In particular, we are able to deduce dynamical regimes under which multistable patterns of neural activity emerge. Importantly, these findings suggest a role for brain operation just beyond criticality to promote cognitive flexibility.

## Introduction

Through its multiscale and interconnected nature, supporting a wide variety of emergent dynamical phenomena, the human brain may be naturally described using techniques from network science. In such a description, network nodes typically describe populations of neurons, grouped according to anatomical region, and edges correspond to white-matter tracts describing large-scale brain ‘structural connectivity’; less commonly, such a network can be used to describe within-cortex connections between cortical columns, for example^1–3^. Such network models have been extensively studied, both from the point of view of their topological properties (see, for example^4–7^, and reviews^8^ and^9^) and, somewhat more recently, in terms of the dynamical activity that such structures support. The latter is of particular pertinence to understanding higher brain function, that being underpinned by a vast dynamical repertoire taking place on a relatively static anatomical substrate. These so-called ‘functional connections’ are variously obtained experimentally via functional magnetic resonance imaging (fMRI), magnetoencephalography (MEG) or electroencephalography (EEG) and describe the correlated neural activity between areas; such networks are interpreted in terms of the putative association of physiological activity in anatomical regions and brain (dys)function. For example, inter-regional coherence is hypothesised to underpin communication and information transfer among distributed brain areas, this idea being popularised as ‘communication-through-coherence’^10,11^. Important examples of well-studied functional networks include resting-state networks such as the ‘default mode network’^12–14^ as well as other attention-based ‘core’ networks^15,16^, and analyses of both structural and functional networks have provided biomarkers for a range of neurological disorders (see, e.g.,^17,18^ for a review), while regional variability in structure–function similarity is highlighted in^19^, and^20^ reports remodelling of these relationships during development, associated with functional specialization and cognition. An overarching question in neuroscience, therefore, is how does brain function arise from the underlying structure? This is a highly active area of research (see^21,22^ for recent reviews), though the precise mechanisms that link structure to function remain unclear. In tandem with such empirical studies are mathematical and computational approaches that employ simulated neural activity together with anatomical data to investigate the structure-function relationship in detail^23–31^. Here, graph theoretical properties of simulated functional networks are complimented by computational and mathematical analyses of the dynamical system at the node- or network-level to quantify the role that local neural dynamics has in shaping functional brain states, and the network’s response to stimulation.

A key feature of empirically-observed neural activity is its dynamic and frequency-specific nature; for example, activity in the $\beta$-band (13–30 Hz) has long been associated with movement, while $\alpha$-band (8–12 Hz) is thought to be fundamental to cognitive processing during wakefulness. A thorough overview of these brain ‘rhythms’ is provided in^32^ (we note in passing that the separation of neural activity into arbitrary frequency bands is not of itself meaningful; indeed, cross-frequency interactions are a well known feature; see, e.g.,^33^ for a review). Nonetheless, together with the various modalities employed in neuroimaging studies, these observations motivate exploiting relatively recent theoretical advances in complex networks that employ multilayer networks to describe multiple interacting network structures simultaneously to interrogate neuroimaging phenomena. For example^34^, consider multilayer networks arising from (frequency-filtered) MEG and fMRI data. An excellent review of multilayer networks employed in a neuroimaging context is given in^35^ and comprehensive reviews of multilayer networks can be found in, e.g.^36–38^.

Of particular relevance to the structure-function issue is the study of^25^, in which a clustering measure is proposed that characterises the emergence of functional connections between brain regions in the absence of direct anatomical links. In particular, such clustering in an unweighted duplex network, comprising a directed structural layer (describing white matter connections in the macaque cortex) and a functional layer derived from simulated neural activity, is analysed. Such a measure provides a powerful metric that reflects the subtle interaction between brain structure and neural dynamics and highlights in particular that such features are better captured by an integrative approach rather than traditional single-layer analyses. However, as is relatively common in the network sciences, and recent developments in multilayer networks in particular, such a measure relies on somewhat arbitrary binarisation choices, that convert dense, weighted functional networks to sparse, binary structures. Such an approach not only omits potentially important information encoded in the heterogeneity of network weights, but omits weak connections in particular, whose contribution to network dynamics in brain (and other) networks is well-known^39–41^. Moreover, concentrating on two-layer multiplexes, the variety of functional network descriptions available from the different neuroimaging modalities or frequency band analyses are neglected.

In this paper, we address these issues to generalise multiplex clustering to describe weighted multiplexes with arbitrarily-many layers, and the aforementioned structure-function clustering to the weighted case. To demonstrate its applicability to the structure-function question of interest, we then adopt an *in silico* approach (similarly to the related studies of^24,25,27^), combining human connectome data with simulated neural population activity. This allows us to explore the application of multiplex network properties to simulated neural dynamics in different dynamical states, which are readily computed via variation of model parameters. Thereby we highlight both the link between criticality (as measured by constructing the model bifurcation sets) and SC-FC similarity, and the divergence of these networks (as exposed by our new measure) beyond these critical points. Our results highlight a region in parameter space beyond criticality in which a large set of functional states are accessible (in contrast to the highly constrained function observed near bifurcation). This suggests an optimal operating space in which the dynamic and flexible repertoire of higher brain function is naturally supported.

## Methods

### Multiplex networks

We represent a multiplex network using the vector $\displaystyle {\mathscr {W}}$ $= \left[ W^{[1]},\ldots ,W^{[M]}\right]$ whose entries $\displaystyle W^{[\alpha ]}=\{w_{ij}^{[\alpha ]}\}\in {\mathbb {R}}^{N\times N}$ are matrices with non-zero entries in the *ij*th position if node *i* connects to node *j* in layer $\alpha$, otherwise $w_{ij}^{[\alpha ]}=0$. In this work we consider undirected multiplexes with normalised weights $0\le w_{ij}^{[\alpha ]} \le 1$ between nodes *i* and *j*. The generalised degree vector, ${\mathbf {k}}$, naturally extends the notion of generalised network degree (or strength) to the multiplex setting such that its entries $k^{[\alpha ]}_i = \sum _jw_{ij}^{[\alpha ]}$ give the generalised network degree of node *i* restricted to layer $\alpha$.

### Weighted multiplex clustering

Recall that for a single layered network the local clustering coefficient of the *i*th node is given by the formula $\displaystyle c(i) = 2t_i/\left[ k_i(k_i-1)\right]$^42^, where here $t_i$ counts the number of triangles including node *i* and $k_i$ denotes its degree. Now, using the fact that powers of the adjacency matrix, $A^k$, count walks of length *k* in the network, we can rewrite the above formula as

1 $$\begin{aligned} c(i)= & {} \frac{\sum _{j}\sum _{k, k\ne j}a_{ij}a_{jk}a_{ki}}{\sum _{j}\sum _{k, k\ne j}a_{ij}a_{ki}},\end{aligned}$$

2 $$\begin{aligned}= & {} \frac{\left( A^3\right) _{ii}}{k_i^2 - \left( A^2\right) _{ii}} = \frac{\left( A^3\right) _{ii}}{k_i(k_i-1)}. \end{aligned}$$

A single global clustering coefficient is then obtained by averaging the above over all nodes.

The formula (1) directly extends to the weighted case^43^:

3 $$\begin{aligned} c_\mathrm {w}(i) = \frac{\sum _{j}\sum _{k, k\ne j}w_{ij}w_{jk}w_{ki}}{\sum _{j}\sum _{k, k\ne j}w_{ij}w_{ki}}. \end{aligned}$$

Note that the numerator gives the strength of triangles involving node *i* and the denominator the strength of the pairs of neighbours involving node *i*. As before, the above formula can be rewritten as

4 $$\begin{aligned} c_\mathrm {w}(i) = \frac{\left( W^3\right) _{ii}}{k_i^2 - \left( W^2\right) _{ii}}, \end{aligned}$$

where here, $k_i$ denotes the weighted network degree of the *i*th node. Importantly, the above measure lies in the range [0, 1] and reduces to the usual formula (2) when the weights are binary.

Now, in a similar vein one can write down a weighted version of the multiplex clustering from Battiston et al.,^44^, Eq. 22, as

$$\begin{aligned} C_{\mathrm {wm}}(i) = \frac{\sum _{\alpha }\sum _{\alpha '\ne \alpha }\sum _{j,k}\left( w_{ij}^{[\alpha ]}w_{jk}^{[\alpha ']}w_{ki}^{[\alpha ]}\right) }{\left( M-1\right) \sum _{\alpha }\sum _{j\ne k}\left( w_{ij}^{[\alpha ]}w_{ki}^{[\alpha ]}\right) },\end{aligned}$$

where $\alpha$ and $\alpha '$ are two distinct network layers. As previously, we can simplify the above to

5 $$\begin{aligned} C_{\mathrm {wm}}(i) = \frac{\sum _{\alpha }\sum _{\alpha '\ne \alpha }\left( W^{[\alpha ]}W^{[\alpha ']}W^{[\alpha ]}\right) _{ii}}{\left( M-1\right) \sum _{\alpha }\left( k_{i}^{[\alpha ]2} - \left( W^{[\alpha ]2}\right) _{ii}\right) }.\end{aligned}$$

Recall that in the above we are assuming that all layers are weighted such that ${w^{[\alpha ]}}_{ij}\in \left[ 0,1\right]$ for $i,j = 1,\ldots N$ and $\alpha = 1,\ldots M$ and that each layer is undirected, i.e. ${W^{[\alpha ]}} = {W^{[\alpha ]T}}$ for all $\alpha =1,\ldots ,M$.

### Weighted structure-function clustering

To further probe structure-function relationships in the brain, the authors in^25^ proposed a modification of the standard multiplex clustering coefficient^44^, in the case of an unweighted two-layer structure-function multiplex, as follows

6 $$\begin{aligned} C_\mathrm {sf}(i) = \frac{\# \text{ structure-function } \text{ triangles }}{\# \text{ structural } \text{ tuples } - \# \text{ structural } \text{ triangles }}. \end{aligned}$$

Here, a structural tuple refers to two structural edges meeting at a common node (i.e. a 3-tuple) and a structure-function triangle comprises such a structural tuple, closed by a functional edge. We subtract the number of structural triangles in the denominator since we are interested in functional connections that arise between structurally unconnected regions (see^25^ for further details).

Mathematically, Eq. (6) can be written as

7 $$\begin{aligned} C_{\mathrm {sf}}&= \frac{\sum _j\sum _{k, k\ne j}a_{ij}^{[1]}a_{jk}^{[2]}a_{ki}^{[1]}(1-a_{jk}^{[1]})}{\sum _j\sum _{k,k\ne j}a_{ij}^{[1]}a_{ki}^{[1]}(1-a_{jk}^{[1]})},\nonumber \\&= \frac{\left( A^{[1]}\left( A^{[2]}\circ \left( I-A^{[1]}\right) \right) A^{[1]}\right) _{ii}}{k^{[1]}_i(k^{[1]}_i-1)(1-c^{[1]}_i)}, \end{aligned}$$

where $\circ$ denotes element-wise multiplication, and $k^{[1]}_i$ and $c^{[1]}_i$ denote respectively, the standard network degree and clustering coefficient of the *i*th node in layer one (i.e. the structural layer). Note that the denominator in (7) follows from rearrangement of the standard single-layer clustering coefficient (1).

As for the standard multiplex clustering coefficient, the equation in (7) naturally extends to weighted networks as follows

8 $$\begin{aligned} C_\mathrm {wsf}(i)&= \frac{\sum _j\sum _{k, k\ne j}w_{ij}^{[1]}w_{jk}^{[2]}w_{ki}^{[1]}(1-w_{jk}^{[1]})}{\sum _j\sum _{k,k\ne j}w_{ij}^{[1]}w_{ki}^{[1]}(1-w_{jk}^{[1]})},\end{aligned}$$

9 $$\begin{aligned}&=\frac{\left( W^{[1]}\left( W^{[2]}\circ \left( I-W^{[1]}\right) \right) W^{[1]}\right) _{ii}}{\left( k^{[1]2}_i-\left( W^{[1]2}\right) _{ii}\right) \left( 1-c_\mathrm {w}^{[1]}(i)\right) }, \end{aligned}$$

where $c_\mathrm {w}^{[1]}(i)$ denotes the weighted clustering coefficient, as given by Eq. (3), of the *i*th node in layer one. As before, all weights are assumed to lie between zero and one.

In all cases, the average clustering coefficient across a multiplex network of *N* nodes per layer is obtained via ${\mathscr {C}}_{\#} = (1/N)\sum _i C_{\#}(i)$ where $\#\in \{\mathrm {wm},\mathrm {sf},\mathrm {wsf}\}$.

### Jaccard similarity

The extent to which structural connectivity overlaps with functional connectivity is captured using the Jaccard similarity index, which for weighted connectivity matrices is given by:

10 $$\begin{aligned} J(W^{[\alpha ]},W^{[\alpha ']})=\sum _i\sum _j\frac{\min (w^{[\alpha ]}_{ij},w^{[\alpha ']}_{ij})}{\max (w^{[\alpha ]}_{ij},w^{[\alpha ']}_{ij})}. \end{aligned}$$

The above ratio provides a natural measure of structure-function similarity, ranging from zero to one, with higher scores denoting greater similarity between the SC and FC matrices.

**Figure 1:**
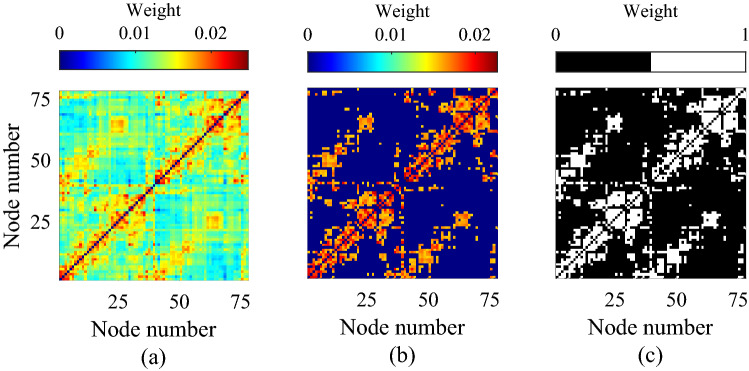
The structural matrix (**a**) is derived from DTI data taken from the Human Connectome Project and parcellated on to a 78–region brain atlas. This is thresholded to keep the top 23% strongest connections (**b**) and binarised by setting all retained edge strengths to unity in (**c**).

### Data

The structural connectivity (SC) is informed by anatomical data obtained from the Human Connectome Project, comprising the average of ten MRI datasets (see^45^ for more detail). This data describes interactions between $N=78$ parcellated brain regions, providing an undirected (symmetric), weighted matrix whose elements $a_{ij}$ indicate the ‘strength’ of interaction between nodes (or brain regions) *i* and *j*. To obtain weights lying in the range [0, 1] we normalise the weight $a_{ij}$ by dividing by the product $\sqrt{k_i k_j}$, where $k_i$ is the generalised degree of node *i*. Thus we obtain a weighted adjacency matrix *W* given by

11 $$\begin{aligned} W = D^{-1/2}AD^{-1/2}, \end{aligned}$$

where the diagonal degree matrix $D\in {\mathbb {R}}^{N\times N}$ has the form $D:=\mathrm {diag}(k_i)$. Note that this normalisation has previously been shown to be beneficial when computing network statistics of anatomical networks derived via MRI^46,47^, where the weights are often poorly calibrated leading to an undue influence of highly promiscuous nodes with large weights. Our primary aim is to demonstrate the applicability of our new weighted clustering measure (9); however, for comparative purposes, two additional descriptions of the cortical network are also considered: (i) a thresholded representation combining topological information with the weight distribution of the network, which was obtained by setting to zero all but the top 23% of values in the matrix *W*, whilst preserving the weights of the remaining edges; and (ii) a binary matrix which was obtained by setting to unity all the edges that survived the thresholding procedure. See Fig. 1 for an illustration of the three different cortical connectivity matrices. Such thresholding and binarisation choices vary throughout the literature; however, we remark that choices made above are consistent with^26^ (itself informed by^48^) and we note that analysis therein demonstrates that these do not unduly influence SC network structure and nor do they qualitatively alter SC-FC correlation structure (albeit employing a different neural mass model). Irrespective, the consequences of these, or alternative, procedures lie outside our focus as (9) specifically treats the fully-weighted network without modification.

### Large-scale neural dynamics

To model large-scale neural dynamics, we consider a network of *N* interacting neural populations, representing a parcellation of the cerebral cortex, such that each network node corresponds to a functional unit that can be represented by a neural mass model, and with coupling between units defined by anatomical structural connectivity data as described in “Data” section. To simulate neural population activity, we employ the Wilson–Cowan model^49^, which describes the dynamics of excitatory and inhibitory neural populations within each node. This simple, but historically important, neural mass model has been widely used to investigate a wide range of neural phenomena, including the structure-function questions of interest here (see the Journal of Neurophysiology Collection ‘50 Years of Modeling Neural Activity: Celebrating Jack Cowan’s Career’^50^ for extensive historical background, and new and future developments influenced by this model) and is defined by the following 2*N* nonlinear ordinary differential equations:

12 $$\begin{aligned} \frac{\mathrm {d} u_i}{\mathrm {d} t}= & {} -u_i + f\bigg (c_1u_i - c_2v_i + P + \epsilon \sum _{j=1}^N b_{ij}u_j\bigg ),\end{aligned}$$

13 $$\begin{aligned} \frac{\mathrm {d} v_i}{\mathrm {d} t}= & {} -v_i + f(c_3u_i - c_4v_i + Q). \end{aligned}$$

**Figure 2:**
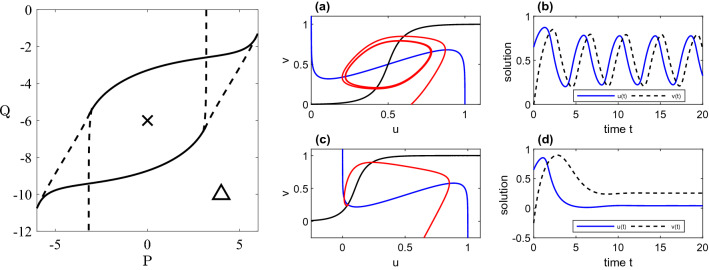
Bifurcation sets and numerical simulations for a single Wilson–Cowan node (12), (13) with $\displaystyle c_1=c_2=c_3=10$ and $\displaystyle c_4=-2$. LHS: The dashed curve denotes the saddle-node bifurcation set and the solid curve the Hopf bifurcation set. RHS: (**a**, **b**) and (**c**, **d**) display phase plane and time courses for oscillatory and steady state solutions (for parameter values indicated by the cross and triangle in the left-hand panel) of Eqs. (12), (13) with $\epsilon =0$.

Here, $u_i(t)$ denotes the activity of the population of excitatory neurons within region *i*, and $v_i(t)$ the activity of inhibitory neurons. The population firing rate is given by the sigmoidal function

$$\begin{aligned} f(x) = 1/\left( 1+\exp {(-x)}\right) ; \end{aligned}$$

interconnectivity is encoded by the adjacency matrix $b_{ij}$, which is given by one of the three connectivity matrices defined in “Data” section. The global between-node connectivity strength is controlled by the parameter $\epsilon$ which we set to unity for the two weighted networks and inversely proportional to the mean degree (i.e. $\displaystyle \epsilon = 1/\langle k\rangle$) for the binary network, thus guaranteeing the input to each node is of the same order for each network. Note that for $\epsilon =0$ the network decouples, with node dynamics determined only by the parameter values and initial data. The constants $c_1,\ldots ,c_4$ define the strength of within-node interactions between neural populations and are chosen to be $c_1=c_2=c_3=10$ and $c_4=-2$ as in^51^, these values being selected to generate oscillatory rhythms in biophysical frequency ranges. The remaining parameters, *P* and *Q* represent the basal input to each population and which we vary as control parameters in order to explore different dynamical states in the investigations that follow. The bifurcation structure of (12), (13) describing parameter sets at which oscillatory solutions are generated for the uncoupled case ($\epsilon =0$) is shown in Fig. 2. These Hopf and saddle-node sets are straightforwardly computed in closed form (see^51^, wherein extensive consideration of the model dynamics under variation of its parameters is given). In the case of the larger networks of interest here we construct bifurcation diagrams describing destabilisation of the uniform steady-state by direct numerical evaluation of the eigenvalues of the linearised system of 2*N* ODEs at (*P*,*Q*) pairs within the explored parameter space, obtained by means of the fsolve and eig routines in MATLAB. Very similar structures to those for the single-node case are obtained, highlighting the role of individual node dynamics on network behaviour, and these are superimposed on the relevant results obtained from direct simulation of (12), (13) presented in “Results and discussion” section. We note in passing that if the dynamics naturally support synchronous solutions (as is the case for networks with a row-sum constraint), we may employ the quasi-analytic approach described in^26^ to obtain the network bifurcation sets in which diagonalisation of the linearised problem in the basis of eigenvectors of the structural connectivity leads to a set of *N* uncoupled two-dimensional problems that can straightforwardly be analysed.

More sophisticated neural mass models that more accurately reproduce EEG or fMRI data^52^ or accommodate important aspects of within-population neural biophysics and emergent synchrony properties^53^ are available, and indeed have recently been deployed to study questions of structure-function relationship^26,28^. However, we restrict attention to a simpler representation as our focus here is to present a new metric for the interrogation of structure-function relations, rather than on the comprehensive study of neural population dynamics themselves.

### Functional connectivity

Functional connectivity (FC) is obtained by direct simulation of the neural mass network defined by (12), (13) using the structural connectivity data described in “Data” section. A small amount of white noise with variance $\sigma = 0.01$ was added to the *u* variable of each node and the system integrated for a total time $T=2000$ using the Euler–Maruyama method, with a time step $\mathrm {d}t = 0.01$. The value of *T* was chosen to ensure that the simulated FC we obtain has converged to a relatively steady pattern. Pairwise synchronisation between the time-series activity of each cortical area is assessed by the Pearson correlation coefficient to provide a matrix describing the strength of functional connection between each region. Note that the period $t<1000$ is discarded in the functional connectivity analysis to allow for initial transients. Functional connectivity matrices were thresholded such that the density of the resulting network was equal to that of the structural connectivity network deployed in the simulation. In the case of the binary structural network the weights in the thresholded FC matrix were additionally set to one.

We note that of course a plethora of functional connectivity measures are available (see, for example, the review by Wang et al.^54^) and that more advanced measures may be employed to derive a directed “effective connectivity” structure^55,56^, or to evaluate the dynamical features of such functional connections^57,58^; however, Pearson’s correlation remains a popular choice for estimating FC in both empirical and modelling studies and so we adopt this to derive time-averaged, undirected functional networks for consistency with similar studies in the literature, and correspondence with^25^, in particular.

**Figure 3:**
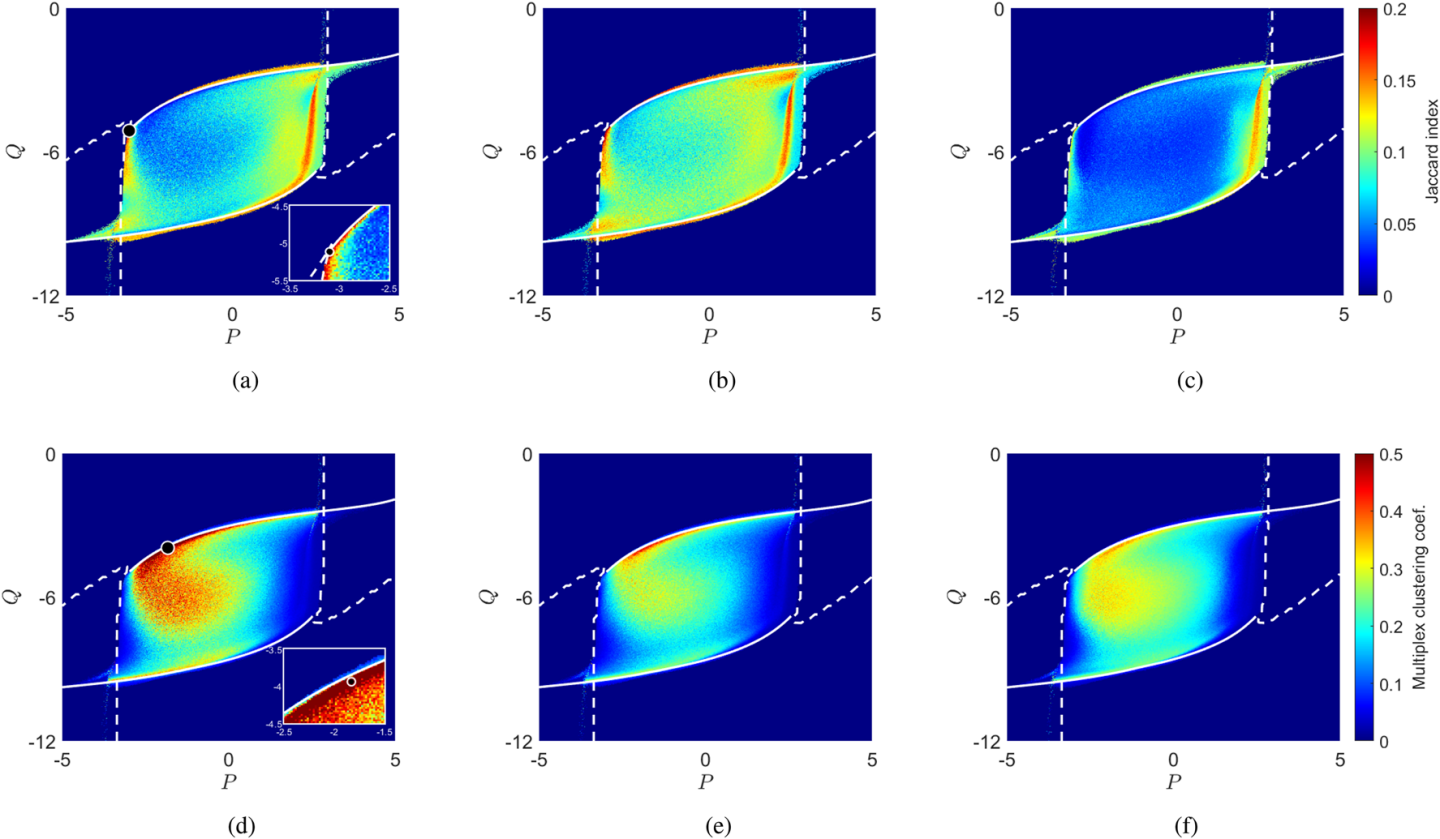
Comparison of the Jaccard index (top row) and structure-function clustering (bottom row) as a function of the basal input parameters (*P*, *Q*) for the three different cortical representations considered in our investigations: (**a**,**d**) weighted network; (**b**,**e**) weighted topological network; and (**c**,**f**) binary network. Bifurcation sets for each network, obtained as described in “Large-scale neural dynamics” section are superimposed in white; the dashed curve denotes the saddle-node bifurcation set and the solid curve the Hopf bifurcation. Specific parameter choices $(P,Q)=(-3.1,-5.12)$ and $(-1.83,-3.94)$ explored in further detail in Figs. 4, 5 are highlighted by the markers in panels (**a**) and (**d**). Insets show their positions relative to the boundaries of the region of interest and regions of high and low structure-function similarity and clustering.

## Results and discussion

Interdependencies of SC and FC are modelled using a two-layer multiplex brain network of the form

14 $$\begin{aligned} {\mathscr {W}} = \left[ W^{[1]}, W^{[2]}\right] . \end{aligned}$$

Here, $\displaystyle W^{[1]} = \{w^{[1]}_{ij}\}$ represents the weight of the physical connection between nodes *i* and *j*. This anatomical layer takes one of the three forms discussed in “Data” section: (1) a fully-weighted network; (2) a ‘weighted topological network’; and (3) a fully binarised network. The functional layer $W^{[2]} = \{w^{[2]}_{ij}\}$ represents the strength of correlations between nodes *i* and *j*, as described in “Functional connectivity” section.

Additionally, to discern whether observed structure-function network patterns are a direct consequence of SC-FC topology, we generated multiplex null models in which the structural layer (layer 1) was obtained by randomising the anatomical network using the Maslov–Sneppen algorithm (or a variant thereof in the weighted case—see the Supplementary Information for further details) and the functional layer (layer 2) was constructed by solving (12), (13) as described in “Functional connectivity” section, but with the connectivity matrix, $B = \{b_{ij}\}$, given by the randomised structural layer.

Figure 3 compares SC-FC similarity, measured by Jaccard index (10) (panels a–c), against structure-function clustering ${\mathscr {C}}_{\mathrm {wsf}}$ (panels d–f), given by Eq. (9) for these multiplex brain structures, as a function of the basal input parameters *P* and *Q*. We see that for all cases, structure-function similarity and clustering are elevated in a region of parameter space whose boundary closely matches the bifurcation structure of (12), (13) that pertains to each network under consideration. In particular, and in concord with empirical and computational studies^59–62^, strongest similarity between structural and functional networks is observed at boundary of the region where the system exhibits critical (i.e. bifurcation) points (Fig. 3a–c). This is especially so for the binary case (Fig. 3c), while richer structures are observed in the interior when weighted information is included; in particular, in the fully weighted case, a curved area of especially low structure-function agreement that emanates from the upper branch of the region appears in the interior, this structure being only weakly visible in the other network structures we study.

In contrast, and in all cases, elevated structure-function clustering reflects less tightly the criticality structure (Fig. 3d–f). In particular, we observe highest clustering for parameter values just beyond creation of oscillatory solutions, and within the curved area in the interior identified above, these features being most pronounced in the fully-weighted case but are evident in all networks considered. High clustering is typically associated with low structure-function similarity (and *vice versa*, as is to be expected), but also includes detailed variation beyond the complement of Fig. 3a–c as one traverses the parameter space.

**Figure 4:**
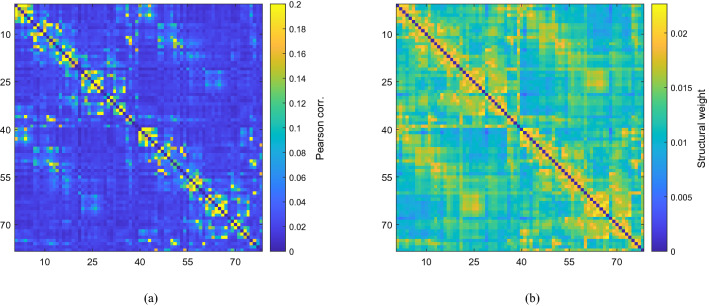
For (*P*, *Q*) values close to criticality the simulated functional connectivity (**a**) is found to resemble the empirical structural connectivity (**b**). The displayed functional connectivity network was obtained by averaging 100 simulations for specific parameter values $(P, Q) = (-3.10, -5.12)$, which are highlighted by a circle in Fig. 3a; the corresponding Jaccard index is 0.195, and across realisations we observe maximal deviation in this value of $\sim 13\%$.

**Figure 5:**
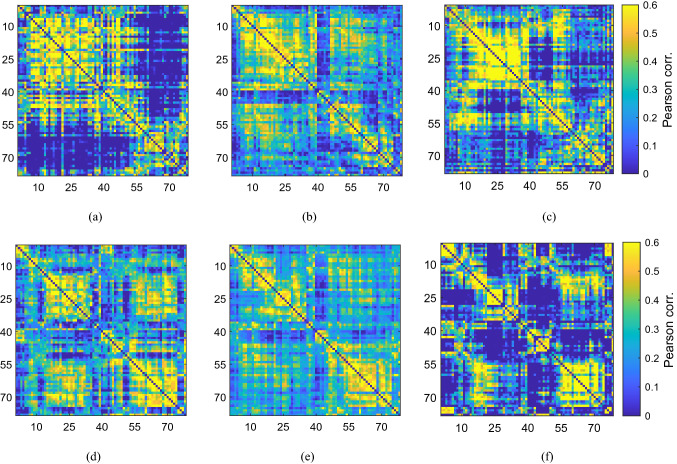
Simulated functional connectivity matrices deploying the weighted structural network with specific parameter values $(P, Q) = (-1.83, -3.94)$, which are highlighted by a circle in Fig. 3d. For these parameter values the network is capable of attaining a range of functional configurations, (**a**–**f**), under variation of the initial conditions. For these parameter values the network is capable of attaining a range of functional configurations under variation of the initial conditions. Across 100 realisations, we observe deviations in the Jaccard index of $50\%$ around a mean value $\sim 0.02$.

We investigate the relationship between structure and function highlighted above in more detail in Figs. 4 and 5. For parameter values on the boundary of the region of interest, at which oscillatory solutions are generated, we observe strong similarity between simulated FC and underlying structure irrespective of the initial state, as expected (see Fig. 4a, which corresponds to the parameter choice highlighted in Fig. 3a). Specifically, the mean Jaccard index is 0.195, with maximum deviation of $\sim 13\%$ across realisations; correspondingly, mean SC–FC clustering is low (${\mathscr {C}}_{\mathrm {wsf}}=0.085$) and with very low spread across realisations (standard deviation $\sim 5.7\%$). In stark contrast, for parameter choices just beyond bifurcation that are identified in Fig. 3d as displaying high weighted structure-function clustering, indicative of divergence between structure and function, we observe a wide variety of functional states that are distinct from the underlying structure, depending on the initial state and additive noise chosen. Some indicative examples are shown in Fig. 5. Here, we observe significant deviations in Jaccard index, reflecting the variety of states accessed ($50\%$ deviation around a low mean value of $\sim 0.02$) and high mean SC–FC clustering (${\mathscr {C}}_{\mathrm {wsf}}=0.67$), though with little variation across realisations (standard deviation $\sim 4.8\%$). We remark in passing that we observe variation in SC–FC clustering $C_\mathrm {wsf}(i)$ across the network (a range of [-40.9%,61.5%] of the mean for the parameter set indicated in Fig. 3a, and [-13.6%,10.9%] for that of 3d), reflecting the empirical findings of^19^ and^20^ discussed previously, but we do not pursue this further here.

We repeated the above analysis on null model ensembles for each of the three multiplexes (i.e. layer 1 given by either a weighted, thesholded or binary network). For randomised binary and thresholded multiplexes, we observed elevated and approximately uniform structure-function similarity, and markedly reduced structure-function clustering, within the region enclosed by the critical branches, in comparison with empirical multiplexes (see Figs. 2, 3 in the supplementary material). In the weighted case, elevated structure-function similarity was observed (to a lesser extent) along the branches of criticality, with a significantly enlarged (in comparison to the empirical multiplex) region of structure-function clustering emanating from the upper critical branch (see Fig. 4 in the Supplementary Material). However, unlike the increased structure-function clustering levels observed for the empirical multiplex, which are a manifestation of cortical network flexibility, as evidenced by the functional connectivity patterns displayed in Fig. 5, the increased levels observed for the random weighted multiplexes arise from the type of excessive synchrony (see Fig. 5 in the Supplementary Material for some example FC matrices) that is often reported for single-layered, weighted networks^63,64^. Importantly, these results indicate that connectome topology may play an important role in both supporting network flexibility and suppressing the type of hyperactivity reminiscent of neurological disorders such as epilepsy.

These observations are of relevance to the neuroimaging application that is our focus here. In view of the relatively static structural substrate on which neural activity operates, it seems clear that divergence of functional activity from this underlying network is fundamental to the brain’s flexible and dynamic repertoire of higher brain function. See, for example^56^, for a review of the network science and neuroscientific aspects of the structure-fuction divergence issue. The preceding results highlight that at criticality, the model dynamics are highly constrained by structure; in contrast, we have identified an operating region beyond criticality in which such flexible brain operation is naturally promoted: here, a dense and highly variable set of distinct functional states are easily accessible, thereby supporting the necessary ability to switch easily between a variety of states that allows for flexible higher brain function.

Our results hence contribute to the debate surrounding the relevance of criticality in neural dynamics, contrasting with the idea that structure-function relations can largely be understood in terms of criticality; see, for example^65,66^, for reviews and discussion.

## Conclusions

In this paper we present an extension of the multiplex clustering coefficient to describe weighted multiplexes with arbitrarily-many layers. A natural application of our developments arises in neuroimaging research, in which many-layered network structures arise naturally both from the variety of imaging modalities employed and the commonly-employed frequency-specific data analyses undertaken. With this context in mind, we additionally extend our previously developed clustering measure^25^ that characterises functional connectivity arising in the absence of structural links to the weighted case.

Our numerical experiments highlight that, as in the binarised case (and reported elsewhere^25,61,67–70^) high structure-function similarity in the weighted case is associated with criticality of the underlying neural model, from which the functional networks are derived, although the addition of weighted information does lead to a far richer structure-function similarity landscape. Structure-function clustering, on the other hand, is not confined to the criticality structure, with highly clustered networks being observed far from bifurcation; highest values are observed in a region just beyond criticality, where structure-function similarity is low. In this region we additionally identify a wide array of functional networks that are accessible (their availability being understood to be a result of multi-stability of network states occurring in regimes beyond criticality), pointing to an operating region in which dynamical switching between brain functional states is naturally promoted. This stands in stark contrast to the system dynamics near bifurcation, at which functional states are strongly constrained by network architecture. Our results hence conform to findings of^71^, which have shown empirically that the resting-state of the brain exhibits stable departures from an anatomically-constrained FC. Indeed, a current focus of computational neuroscience is to define mechanisms by which the brain switches between different brain states (see^72^ for a recent review), and our work contributes to this topic by offering a novel method through which we have been able to identify dynamical regimes amenable to a variety of functional brain states.

In summary, in our newly developed clustering measure, we provide a potential diagnostic tool with which to interrogate the rich information contained within multimodal neuroimaging datasets and that hence promises to aid further understanding of brain activity in health and disease. Via *in silico* investigations employing a paradigmatic neural mass model, we demonstrate how such a measure can provide insight into the dynamical landscape of cortical brain states that facilitate higher brain function. Natural future avenues of investigation include deploying our newly-developed measure on empirical structure–function data, and in *in silico* studies of the type presented here that employ more biophysically-faithful models of neural activity (e.g.^28^, and extensions thereof) and of brain connectivity, incorporating important information on network heterogeneity, such as myelination or path-length data to inform connectivity strength, conduction speed and delays^73,74^. Thereby, spatial and temporal variations in structure-function connectivity can be further examined by our new measure, potentially to enhance understanding of brain function and development.

## Supplementary Information

Supplementary Information.

## Data Availability

The data used in this work is freely available from the Connectome Coordination Facility database https://www.humanconnectome.org/. Additional post-processed data and code is available upon request.
