# Supplementary material for: Structure-function clustering in weighted brain networks
Source: Sci Rep. 2022 Oct 6;12:16793. doi: 10.1038/s41598-022-19994-9 (PMC9537289; doi:10.1038/s41598-022-19994-9)
Supplement: Supplementary file 1 — Supplementary Information. [file 41598_2022_19994_MOESM1_ESM.pdf]

Weighted structure-function clustering: Supplementary Information

Jonathan J Crofts, Michael Forrester, Stephen Coombes & Reuben D O'Dea

1 Randomisation algorithm

The random surrogate networks are constructed, in the case of a binary SC matrix, via an algorithm presented in [1]. At each iteration of the algorithm, two pairs of connected nodes, $B \to A$ and $D \to C$, are chosen such that no edges exist from $D \to A$ and $B \to C$. The connections within the chosen pairs are then removed and new connections are added to the pairs $D \to A$ and $B \to C$. This has previously been used in the related studies of [2] and [3] to provide random surrogate networks for binary, directed SC matrices of the macaque monkey. Here, we make amendments to this algorithm in order to treat weighted structural networks (both all-to-all and thresholded). In the following, we describe the randomisation procedure at each iteration of the algorithm, which is generalised for directed networks. This easily extends to preserve symmetry (such that the random matrix is equal to its transpose), by repeating the procedure at each step with subscript indices reversed, *i.e* $w_{AB} \to w_{BA}$. A schematic of the algorithm for (for each network type) provided in Figure 1.

In the threshold case, we again choose two pairs of connected nodes, where $A \to B$ has the smaller of the two connection weights, but enforce the condition that an edge exists from $D \to A$, but not from $B \to C$. Furthermore, we require $w_{AB} < 1 - w_{AD}$, in order to keep all edge weights within $[0, 1]$. If this is satisfied, we assign new edge weights $w^{\star}_{AB} = 0$, $w^{\star}_{CD} = w_{CD} - w_{AB}$, $w^{\star}_{AD} = w_{AD} + w_{AB}$, $w^{\star}_{CB} = w_{AB}$. This ensures that in the 4-node sub-network we have chosen, we have 3 edges pre- and post-randomisation to preserve connection density.

For all-to-all weighted networks, we can alter the algorithm further. Again selecting four random nodes (we are now guaranteed that edges exist between all pairs), we can choose a value X selected (randomly) from a uniform distribution $[0, \min\{w_{AB}, 1-w_{AD}, 1-w_{CB}, w_{CD}\}]$ and again assign new node weights $w^{\star}_{AB} = w_{AB} - X$, $w^{\star}_{CD} = w_{CD} - X$, $w^{\star}_{AD} = w_{AD} + X$, $w^{\star}_{CB} = w_{CB} + X$.

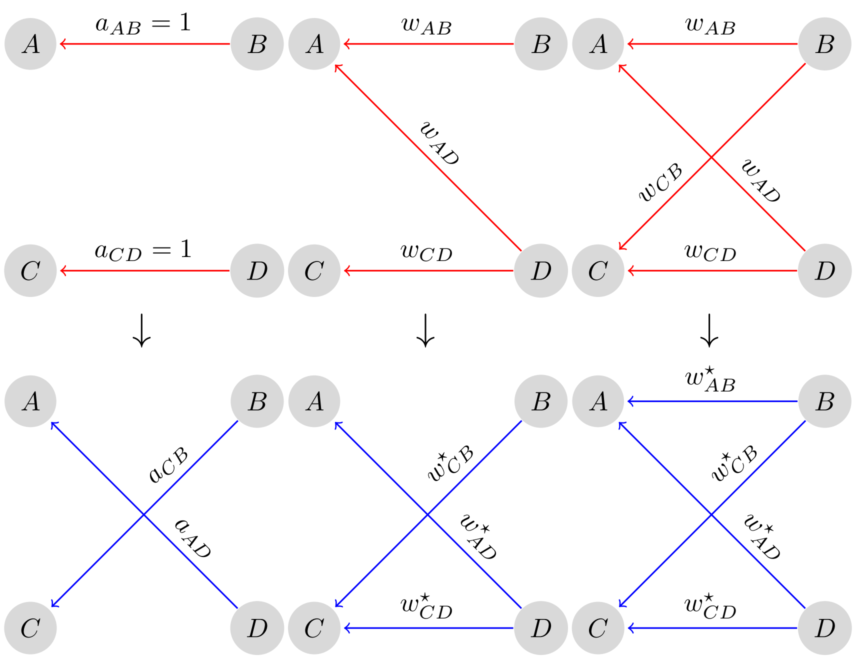

(a) (b) (c)

Figure 1: **Randomisation algorithm schematics.** An illustration of how connections and associated weights change at each iteration of the randomisation procedure for (a) binary thresholded, with $a_{ij} = 1$ if an edge exists from $j \to i$ and 0 if not; (b) weighted thresholded, where w_{ij} is the weight of the edge from $j \to i$ and $w^{\star}_{CD} = w_{CD} - w_{AB}$, $w^{\star}_{AD} = w_{AD} + w_{AB}$, $w^{\star}_{CB} = w_{AB}$; (c) weighted all-to-all networks, with $w^{\star}_{AB} = w_{AB} - X$, $w^{\star}_{CD} = w_{CD} - X$, $w^{\star}_{AD} = w_{AD} + X$, $w^{\star}_{CB} = w_{CB} + X$, $X \in [0, \min\{w_{AB}, 1 - w_{AD}, 1 - w_{CB}, w_{CD}\}]$.

2 Jaccard Index

Jaccard similarity provides a method for assessing similarity between structural and functional layers. In the binary case it is computed as the following ratio

$$J(A^{[1]}, A^{[2]}) = \frac{|A^{[1]} \cap A^{[2]}|}{|A^{[1]} \cup A^{[2]}|}, \tag{1}$$

where, $A^{[1]}, A^{[2]}$ are the adjacency matrices for the structural and functional layers, respectively. This measures the relative number of common SC, FC edges with respect to the total number of edges present in at least one of the adjacency matrices.

For matrices with non-negative real entries the above Jaccard index gener-

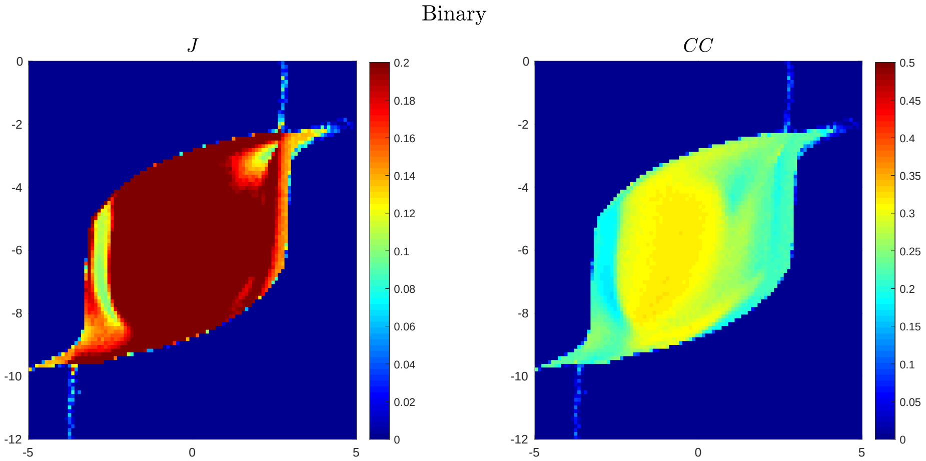

Figure 2: Left: Jaccard index; and Right: multiplex clustering for the randomised binary multiplex. Results averaged over 100 instances.

alises as follows

$$J(A^{[1]}, A^{[2]}) = \frac{\sum_{i,j} \min\left(a_{ij}^{[1]}, a_{ij}^{[2]}\right)}{\sum_{i,j} \max\left(a_{ij}^{[1]}, a_{ij}^{[2]}\right)}.$$

Note that the above formulation reduces to that in (1) when the matrices $A^{[1]}, A^{[2]}$ are binary.

3 Results

Null models were generated using the algorithm described above for each of the three multiplex brain networks, *i.e.* (i) a fully binarised network; (ii) a thresholded network; and (iii) a fully weighted network. We ran 10,000 iterations of the algorithm to randomise the structural layers and simulated dynamics using equations (11), (12) (numbered as in the main manuscript) to produce time-series describing neural dynamics at each node. The functional layer was obtained using Pearson correlation between these time series and thesholded such that the two layers had equal densities. (See the main manuscript for further details.)

3.1 Jaccard and SF Clustering

Jaccard index and structure-function clustering were computed within the same parameter space as in Figure 3 (main manuscript) and averaged over 100 realisations in each instance. The results are shown in figures 2, 3 and 4.

The picture is broadly the same for random networks for which the topology is known in that structure-function similarity is relatively high for all points

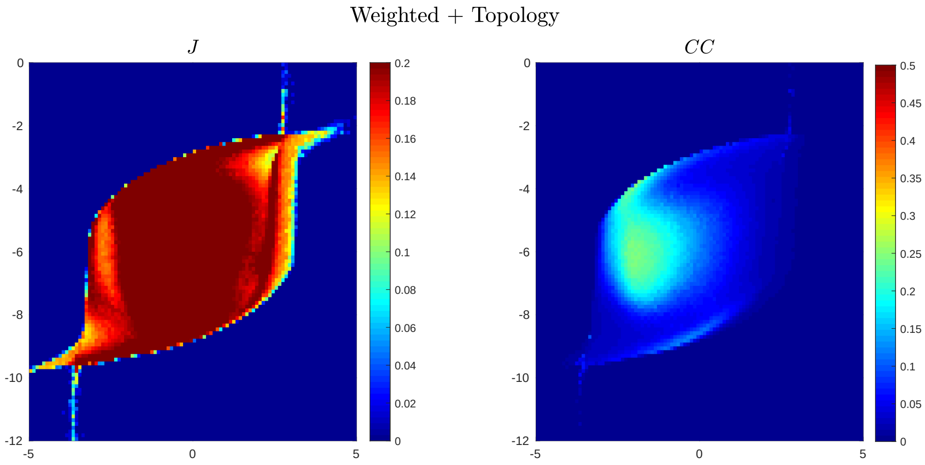

Figure 3: Left: Jaccard index; and Right: multiplex clustering for the randomised thresholded multiplex. Results averaged over 100 instances.

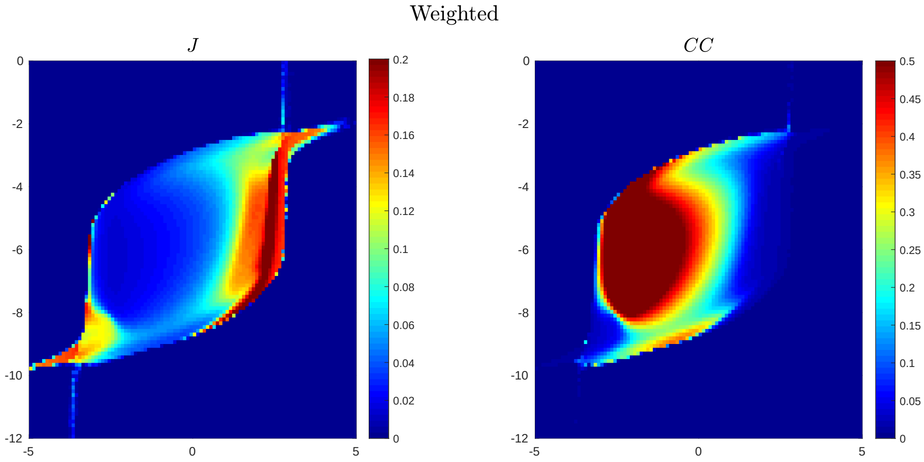

Figure 4: Left: Jaccard index; and Right: multiplex clustering for the randomised weighted multiplex. Results averaged over 100 instances.

within the region bounded by the critical branches; whilst we observe a significant decrease in structure-function clustering throughout. Together these results suggest less flexibility in the randomised structures with function more closely aligning with structure. (See figures 2 and 3.)

For the randomised weighted multiplex, we observe increased structure-function similarity close to the critical boundaries, although not along all of them. Structure-function clustering is increased with a large curved region emanating from the upper critical branch. It is worth noting that the region of increased structure-function clustering observed for the empirical networks is contained within this region. However, unlike the empirical networks, for which increased structure-function clustering is associated with a variety of spatio-temporal patterns akin to experimentally observed resting state networks (RSN), the observed increases in Figure 4 are due to excessive synchronisation.

3.2 Functional connectivity plots

Figure 5 shows representative plots of the functional layer for parameter values chosen from the high structure-function clustering region in Figure 4 (RHS plot). Specifically, we choose $(P, Q) = (-1.83, -3.94)$ which are the same as those chosen in Figure 5 of the main manuscript. As detailed in the previous section, we find that high values of atructure-function clustering are a result of excessive synchrony, in contrast to the empirical multiplex which exhibits a variety of RSN-like patterns.

References

[1] Sergei Maslov and Kim Sneppen. Specificity and stability in topology of protein networks. *Science*, 296(5569):910–913, 2002.

[2] Jaroslav Hlinka and Stephen Coombes. Using computational models to relate structural and functional brain connectivity. *European Journal of Neuroscience*, 36(2):2137–2145, 2012.

[3] Jonathan J Crofts, Michael Forrester, and Reuben D O'Dea. Structure-function clustering in multiplex brain networks. *EPL (Europhysics Letters)*, 116(1):18003, 2016.

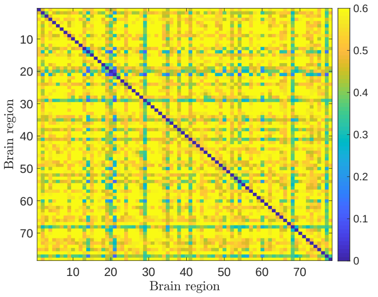

(a)

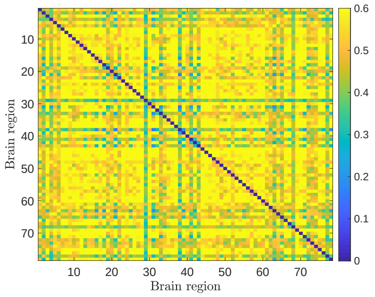

(b)

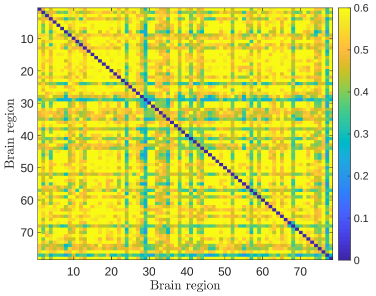

(c)

Figure 5: Simulated functional connectivity matrices (layer 2) deploying the randomised weighted structural network with specific parameter values $(P, Q) = (-1.83, -3.94)$, which are the same as those used in Figure 5 of the main manuscript.
